# Sex differences in self-reported symptoms and comorbidities associated with hypermobile Ehlers-Danlos syndrome and hypermobility spectrum disorders: A retrospective study

**DOI:** 10.21203/rs.3.rs-8310986/v1

**Published:** 2026-01-30

**Authors:** Frances C. Wilson, Derek J. Zangerle, Ashley A. Darakjian, Mira Bhutani, Jessica J. Fliess, Jessica M. Gehin, Max W. Strandes, Charwan Hamilton, Hanna J. Sledge, David O. Hodge, Benjamin W.E. Wang, Paldeep S. Atwal, Ashley M. Zeman, Chrisandra L. Shufelt, Shilpa N. Gajarawala, Katelyn A. Bruno, Dacre R.T. Knight, DeLisa Fairweather

**Affiliations:** Mayo Clinic; Mayo Clinic; Mayo Clinic; Mayo Clinic; Mayo Clinic; Mayo Clinic; Mayo Clinic; Mayo Clinic; Mayo Clinic; Mayo Clinic; Mayo Clinic; Atwal Clinic, West Palm Beach; Mayo Clinic; Mayo Clinic; Mayo Clinic; University of Florida; Mayo Clinic; Mayo Clinic

**Keywords:** hypermobile Ehlers-Danlos syndrome, hypermobility spectrum disorders, comorbidities, sex differences, mast cells, psychology, connective tissue disorders, fibromyalgia, autonomic dysfunction, abuse

## Abstract

**Background:**

Although hypermobile Ehlers-Danlos syndrome (hEDS) and hypermobility spectrum disorders (HSD) are inherited disorders expected to display a 1:1 sex ratio, most studies report a strong female dominance. This study analyzed sex differences in 122 self-reported symptoms and comorbidities in patients diagnosed with hEDS or HSD using the 2017 criteria.

**Methods:**

Self-reported data was obtained from November 1, 2019, to April 25, 2025, (*n*=2,451) from an Intake Questionnaire of adult (≤18 years old) patients diagnosed with hEDS or HSD at the EDS Clinic at Mayo Clinic Florida and analyzed by sex.

**Results:**

We found that 90.6% (*n*=575) of hEDS patients were female and 9.4% (*n*=60) were male, with a sex ratio of 9.6:1 female-to-male, while 95.2% (*n*=1,728) of HSD patients were female and 4.8% (*n*=88) were male, with a sex ratio of 19.6:1 female-to-male. Females were older than males: hEDS 35 vs. 28 years of age (*p*<0.001), HSD 34 vs. 31 years of age (*p*=0.004). Overall, females self-reported more symptoms/comorbidities than males: hEDS 31/122 (25.4%), HSD 59/122 (48.4%). In contrast, only 5/122 (4.1%) symptoms/comorbidities were self-reported more often in males including attention deficit disorder/attention deficit hyperactivity disorder, delay in developmental milestones, snoring, autism spectrum disorder, and obstructive sleep apnea. Females with HSD had more mast cell-related symptoms (i.e., hives *p*<0.001) which were reflected in higher mast cell scores than males (*p*<0.001). Patients with higher mast cell scores also had higher serum levels of total IgE (*p*=0.029). More females with HSD were diagnosed with fibromyalgia (*p*<0.001) and reported symptoms associated with autonomic dysfunction than males. Aside from abuse, which was higher in females (hEDS *p*=0.039, HSD *p*<0.001), few sex differences were reported for psychological symptoms/comorbidities. In support for the idea that elevated mast cell activity in females may lead to extracellular matrix remodeling that promotes hypermobility, females with hEDS/HSD had greater serum collagen-4α1 (*p*<0.0001) and fibronectin (*p*=0.015) than males by ELISA.

**Conclusions:**

Females with hEDS and HSD report a higher burden of symptoms/comorbidities than males, providing a possible explanation for fewer males being reported in demographic data. Sex differences in symptoms/comorbidities may reflect sex differences in pathogenic drivers of disease.

## Plain English Summary

Hypermobile Ehlers-Danlos syndrome (hEDS) and hypermobility spectrum disorders (HSD) are inherited conditions that affect the body’s connective tissues, leading to joint hypermobility, chronic pain, and a variety of other symptoms. Although these disorders should occur equally in men and women, most diagnosed patients are women. This study examined possible reasons for that difference by comparing symptoms and health conditions reported by 2,451 adults diagnosed with hEDS or HSD at the Mayo EDS Clinic in Florida between 2019 and 2025. We found that over 90% of hEDS and HSD patients were female, with a female-to-male ratio ranging from 10:1 to 20:1. Women were slightly older than men and reported more symptoms and associated health problems. Men more often reported only a few conditions, including ADHD, developmental delays, snoring, autism, and sleep apnea. Women with HSD reported more mast cell symptoms, a type of immune cell that releases chemicals like histamine and may contribute to tissue remodeling and hypermobility. These higher mast cell symptoms were associated with more allergy symptoms, like hives, and higher IgE levels in the blood. Women with HSD were also more likely to have fibromyalgia and symptoms of autonomic dysfunction (such as dizziness or heart rate changes). Overall, our findings suggest that biological sex differences- possibly through mast cell activation- may drive differences in disease severity and symptom patterns. This may explain why more women are diagnosed and seek treatment for hEDS and HSD, while men may remain underdiagnosed or less affected.

## Background

Hypermobile Ehlers-Danlos syndrome (hEDS) and hypermobility spectrum disorders (HSD) are connective tissue disorders associated with a wide array of symptoms and comorbidities including joint hypermobility and widespread pain.(1) The hypermobile forms of EDS, hEDS and HSD, are believed to be the most common types of EDS based on survey data.(2, 3) Unlike other forms of EDS, genetic variants leading to hEDS and HSD have not yet been identified; however, both conditions are assumed to involve extracellular matrix (ECM) remodeling due to dysfunction of collagen or other connective tissue proteins.(3) Diagnosis of hEDS and HSD is based on the 2017 diagnostic criteria created by the International EDS Consortium.(4–6) Comorbidities associated with hEDS/HSD include subluxations, dislocations, chronic pain, fatigue, headache, migraine, anxiety, neurological, gastrointestinal (GI) and other symptoms leading to a reduced quality of life.(7–10) Due to the complexity of hEDS/HSD and a limited number of specialists, survey data indicates it can take from 14–28 years to obtain a diagnosis.(11)

We previously reported that around 70% of hypermobile patients seen at our EDS Clinic were also diagnosed with fibromyalgia,(10) a disorder associated with widespread joint and muscle pain and fatigue. Patients that were diagnosed with hEDS or HSD and fibromyalgia self-reported more widespread symptoms/comorbidities than those without a fibromyalgia diagnosis.(10) We also previously reported that hEDS patients self-report more symptoms/comorbidities associated with ECM dysregulation such as dislocations, hernias and prolapses while patients with HSD report more organ or system issues such as joint/muscle symptoms, GI complaints, and neurological symptoms suggesting there may be important pathologic differences between the two diagnoses.(12) Other studies have had similar findings,(10, 12–17) although how much symptom overlap exists between the two diagnoses is being debated.(18) Understanding whether similarities and differences exist are critical as the field considers revising the diagnostic guidelines.

Another factor that may reveal pathogenic drivers of disease are whether sex differences exist in symptoms and comorbidities in hEDS and HSD patients. Although hEDS is an autosomal dominant disorder with an expected sex ratio of 1:1, many studies have shown a 9:1 or greater female-to-male sex ratio indicating that sex differences exist.(12, 19, 20) However, few studies have examined sex differences in symptoms/comorbidities in hEDS and HSD.(21, 22) A recent study by Petrucci et al. examined 23 symptoms and comorbidities by sex in 2,149 patients diagnosed with hEDS.(22) Although the Petrucci et al. study reported symptoms and comorbidities by sex, they did not analyze the data according to sex. In this study, we analyzed data by sex for 122 symptoms/comorbidities in over 2,400 patients diagnosed with hEDS or HSD using the 2017 diagnostic criteria to better understand sex differences in symptoms/comorbidities in the two diagnoses.

## Methods

### Ethics statement

The Institutional Review Board (IRB) of Mayo Clinic approved the retrospective analysis of demographic and clinical data from medical records for this study and waived informed consent for all patients (IRB# 19–011260). The IRB of Mayo Clinic approved the prospective study of samples and informed consent was obtained for all patients (IRB# 19–010260). The research conformed to the principles outlined in the Declaration of Helsinki.

### Primary patient population

A total of 2,451 patients were seen at the EDS Clinic in Mayo Clinic Florida from November 1, 2019, to April 25, 2025, and were used for an analysis of sex differences based on the Intake Questionnaire.(10, 12, 23) Individuals were either self-referred or referred through clinicians within or outside of Mayo Clinic. Diagnosis of hEDS or HSD was established by physician experts using the 2017 diagnostic criteria,(4–6) as previously.(10, 12, 23) Briefly, the diagnostic criteria for hEDS includes identification of generalized joint hypermobility (GJH) of specific joints using the Beighton Scale (past puberty ≥ 5/9 and over 50 years of age ≥ 4/9), evidence of a systemic connective tissue disorder, family history and/or musculoskeletal complications, and several exclusions.(4) HSD is diagnosed in patients that do not meet the diagnostic criteria for hEDS but have a positive Beighton Score (≥ 5/9 and ≥ 4/9 for older patients) and evidence that the joint hypermobility is causing widespread problems and is not just an asymptomatic feature (feature C of the 2nd EDS criterion).(4–6) We did not include patients diagnosed with localized, historical or peripheral HSD in this study.(5, 6, 23)

### Data collection

Adults (≥ 18 years of age) that were diagnosed with hEDS or HSD were included in the retrospective and prospective study. Additionally, patients that were able to sign a consent form were included in the prospective study. Prior to their first appointment at the Mayo Clinic Florida EDS Clinic, patients completed a REDCap Intake Questionnaire as part of their clinical care which asked questions about symptoms and comorbidities in all areas of the body.(10, 12, 23) The REDCap was designed by Drs. Bruno and Fairweather based on patient symptoms. The Intake Questionnaire questions are based on organs or systems like joints, neurological or gastrointestinal symptoms and comorbidities.(10, 12, 23) Data in the manuscript are organized in this manner. A total of 122 of the questions from the Intake Questionnaire were analyzed in this study.

### Mast cell score

**Figure 1:**
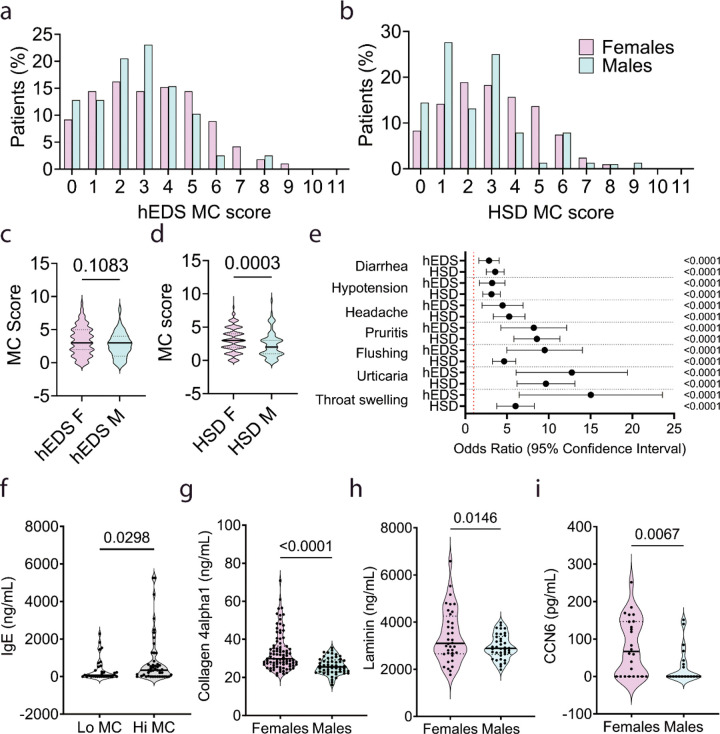
Sex differences in MC score and extracellular matrix proteins in females and males with hEDS and HSD. Distribution of the MC score in females vs. males for patients with a) hEDS or b) HSD. Average MC score showing median and quartiles for females (F) and males (M) with c) hEDS or d) HSD. P values were determined using two-tailed Fisher’s exact tests. e) Odds ratios with 95% confidence intervals comparing hEDS and HSD patients with low (0–1) vs. high (≥5) MC scores for consensus MC activation symptoms.(25) P values were determined using two-tailed Fischer’s exact test. f) Total IgE in serum of patients with hEDS or HSD was examined by ELISA comparing patients with low MC score (Lo MC) vs. high MC score (Hi MC). Data are shown as median ±quartiles using a one-tailed Mann-Whitney rank test (Lo MC, *n* = 37; Hi MC *n* = 43). Human g) collagen4α1 (*n*= 39/sex), h) laminin (*n* = 39/sex), and i) CCN6 (*n* = 25/sex) levels in serum of females vs. males with hEDS/HSD were examined by ELISA. Data are shown as median ±quartiles using a one-tailed Mann-Whitney rank test.

The mast cell (MC) score was newly developed by Drs. Fairweather and Bruno and is based on a range of MC responses (i.e., allergy, atopy, asthma). A higher MC score indicates higher MC activity. In a separate study from the main sex differences analysis, MC symptom data from 1,942 patients were obtained from June 17, 2020, to October 9, 2025, and used to obtain MC scores based on diagnosis and sex. To obtain the MC score patients were asked a series of 11 questions related to categories of MC activity that included: 1. Do you have any environmental allergies? 2. Do you have a venom (i.e., snake bite) or stinging insect (i.e., bee) allergy? 3. Do you have any food allergies? 4. Do you have any drug/chemical allergies? 5. Do you have allergic rhinitis, otherwise known as hay fever? 6. Do you have atopic dermatitis (eczema)? 7. Do you have atopy (a hypersensitivity reaction that occurs in any area of the body that is not in contact with the allergen; often occurs on skin)? 8. Do you currently have or have ever had asthma? Have you been told that you have a hyper immune response like mast cell activation including: 9. Mast cell activation syndrome (MCAS)? 10. Overactive mast cells? 11. Tryptase mutation? A minimum of 10 of the 11 questions were required to be answered for a valid score. Each ‘yes’ answer was given a value of ‘1’ so that the MC score ranged from 0/11 to 11/11 and can be shown as an average ± SD comparing groups, similar to the adverse childhood events (ACE) score.(24) Alternatively, based on the distribution of MC scores by diagnosis and sex (Fig. 1a,b), MC scores of 0–1 were chosen to represent low MC scores (Lo MC) and MC scores of ≥ 5 were chosen to represent high MC scores (Hi MC).

In a separate study, comparisons were made between male and female patients with low vs. high MC scores by diagnosis for symptoms/comorbidities. MC score data from 1,292 patients with high or low MC scores were obtained from June 6, 2020, to June 3, 2025. This analysis included males and females for each diagnosis and was used to generate the odds ratios found in Fig. 1e.

### ELISA

Enzyme-linked immunosorbent assays (ELISAs) were used to determine the level of total IgE, collagen-4α1, fibronectin and cellular communication network factor 6 (CCN6) in the serum of females and males diagnosed with hEDS or HSD. The following human kits were used: total IgE (Invitrogen, catalog #BMS2097, limit of detection 0.5 ng/mL), collagen-4α1 (Antibodies Online, catalog #ABIN1114261, limit of detection 0.31 ng/mL), laminin (Abcam, catalog #ab119599, limit of detection 10 pg/mL), and cellular communication network factor 6 (CCN6) (Assay Genie, catalog # HUFI00701, limit of detection 56.25 pg/mL). The number of patients used per group for each ELISA is listed in the figure legend.

### Statistical analysis

Continuous variables were summarized in tables with the sample mean and range and in figures with mean ± SD. Categorical variables were summarized in tables with number and percentage of subjects. Fisher’s exact tests were used to compare categorical variables between two groups. Student’s *t* test or Mann-Whitney rank test was used to compare continuous variables between two groups. Fischer’s exact test was used to determine odds ratios with 95% confidence intervals. The specific tests that were used are listed in the legends of the tables and figures. Bonferroni correction of the 122 categorical variables in this study indicated that a p-value < 0.0004 was significant. There were no comparisons in this study from the questionnaire with p-values < 0.0004. Considering the numerous symptoms/comorbidities in this study with known sex differences in the general population and during disease, this suggested type II errors. For this reason, data are presented without Bonferroni correction and p-values < 0.05 were considered statistically significant. All statistical analyses were conducted using SAS version 9.4M9, Microsoft Excel and/or GraphPad PRISM 10.6.0.

## Results

### Patient demographics

Of the 2,451 patients who were diagnosed with hEDS or HSD at the EDS Clinic using the 2017 diagnostic criteria,(4–6) we found that 74.1% (*n* = 1,816) were diagnosed with HSD, and 25.9% (*n* = 635) with hEDS. Of the 635 patients diagnosed with hEDS, 90.6% (*n* = 575) were female and 9.4% (*n* = 60) were male with a sex ratio of 9.6:1 female-to-male (Table 1). Of the 1,816 patients diagnosed with HSD, 95.2% (*n* = 1,728) were female and 4.8% (*n* = 88) were male with a sex ratio of 19.6:1 female-to-male (Table 1). We also found that females were older than males for both diagnoses: hEDS 35 vs. 28 years of age (*p* < 0.001), HSD 34 vs. 31 years of age (*p* = 0.004). There were no significant differences in race/ethnicity between males and females with hEDS or HSD, and most patients were White, non-Hispanic (around 90–95%) (Table 1). The highest level of education was significantly different between males and females for HSD (*p* = 0.001) but not for hEDS (*p* = 0.07) (Table 1).

There were no significant differences between females vs. males with hEDS or HSD for BMI, number of cigarettes smoked/day, number of alcoholic drinks consumed on average/week, alcohol exposure before birth, or exposure to drugs as a baby (Table 2). However, we observed sex differences for several other environmental exposures. We found that more male hEDS patients were current smokers (Female 10.1%, Male 17.5%; *p* = 0.005) and illicit drug users (Female 2.6%, Male 8.8%; *p* = 0.036) than females (Table 2). There were no sex differences for current smoking or illicit drug use in HSD patients. However, females with HSD had more current alcohol consumption than males (Female 51.5%, Male 44.8%; *p* = 0.007) (Table 2).

### Joint, muscle weakness and bruising

Joint pain (Female 90.3%, Male 76.7%; *p* < 0.001), sprains (Female 75.1%, Male 50.0%; *p* < 0.001), and easy bruising (Female 56.7%, Male 23.3%; *p* < 0.001) were all self-reported more often in females with hEDS compared to males (Table 3). However, joint pain (Female 94.6%, Male 87.5%; *p* = 0.005), subluxations (Female 76.4%, Male 64.8%; *p* = 0.013), sprains (Female 69.3%, Male 50.0%; *p* < 0.001), easy bruising (Female 64.6%, Male 28.4%; *p* < 0.001), and temporomandibular joint (TMJ) symptoms (Female 49.0%, Male 27.3%; *p* < 0.001) were self-reported more often in females with HSD compared to males. Nothing in this category was reported more often in males than females. Another way to view the data is that 4/7 (57.1%) of these symptoms were the same in males and females with hEDS, while only 2/7 (28.6%) were the same by sex in HSD (Table 3), suggesting that sex differences occurred more often in HSD than in hEDS patients.

### Teeth and jaw symptoms/comorbidities

The presence of a high or narrow palate was the only symptom/comorbidity in this category that showed a sex difference for patients with hEDS (1/7, 14.3%), occurring more often in females than males (Female 31.3%, Male 18.3%; *p* = 0.037) (Table 4). In contrast, sex differences occurred in 6/7 (85.7%) of teeth and jaw symptoms/comorbidities in patients with HSD, occurring more often in females. Nothing in this category was reported more often in males than females. Thus, sex differences occurred more frequently in this category among HSD patients, whereas both males and females with hEDS reported experiencing these symptoms.

### Mast cell-related symptoms/comorbidities

First, we examined whether patients self-reported more mast cell (MC) symptoms based on 11 self-reported symptoms related to allergies, atopy and asthma, for example (see Methods), from the Intake Questionnaire to create an MC score with a range of 0/11 to 11/11. A higher MC score indicates more widespread MC activity. The distribution of answers to each of the 11 MC questions by sex and diagnosis is shown in Fig. 1a,b. We found that there was no sex difference in the MC score in females vs. males with hEDS (Females 3.3 ± 2.1 vs. Males 2.7 ± 1.8, *p* = 0.11) while females with HSD had a significantly higher MC score than males (Females 3.1 ± 1.9 vs. Males 2.4 ± 1.9, *p* = 0.0003) (Table 5, Fig. 1c,d). Thus, females with HSD have evidence of more MC symptoms than males based on 11 common MC symptom categories. In contrast, females with hEDS had a similar level of MC symptoms as males.

To further examine the 11 symptoms that we used to generate the MC score, we compared symptoms in hEDS or HSD patients with a low MC score of 0–1 (Lo MC) to patients with a high MC score of ≥ 5 (Hi MC) with males and females combined. We found significantly higher odds ratios (OR) with 95% confidence intervals (CI) in hEDS and HSD patients with high vs. low MC scores for all MC-related self-reported symptoms that were used to derive the MC score including environmental allergies (hEDS OR 144.3, CI 479.8–1492.0. *p* < 0.0001 ; HSD OR 68.0, OR 116.0–199.0, *p* < 0.0001), venom allergy (hEDS OR 7.6, CI 18.3–43.0 *p* < 0.0001; HSD OR 20.0, CI 47.3–111.9; *p* < 0.0001), food allergy (hEDS OR 20.2, CI 49.2–114.9, *p* < 0.0001; HSD OR 35.4, CI 57.6–92.9, *p* < 0.0001), drug allergy (hEDS OR 19.0, CI 34.1–61.5, *p* < 0.0001; HSD OR 19.9, CI 28.5–40.0, *p* < 0.0001), rhinitis (hEDS OR 43.9, CI 103.5–231.6, *p* < 0.0001; HSD OR 101.6, CI 183.8–327.8, *p* < 0.0001), eczema (hEDS OR 12.2, CI 26.3–54.5, *p* < 0.0001; HSD OR 9.2, CI 13.4–19.5, *p* < 0.0001), atopy (hEDS OR 96.7, CI 456.1–1987.0, *p* < 0.0001; HSD OR 47.0, CI 139.4–350.5, *p* < 0.0001), current or history of asthma (hEDS OR 11.5, CI 24.0–49.3, *p* < 0.0001; HSD OR 28.6, CI 49.7–89.0, *p* < 0.0001), and MC activation syndrome (MCAS) (hEDS OR 6.2, CI 15.0–35.3, *p* < 0.0001; HSD OR 15.9, CI 41.1–106.2, *p* < 0.0001). Data for other MC activation and tryptase mutation are not shown in the graph because of high CIs.

To further validate the MC score, we examined MC activation symptoms that are part of the criteria for a diagnosis of MCAS according to the consensus statement by Valent et al.(25) These symptoms included flushing (hEDS OR 5.3, CI 8.8–14.3, *p* < 0.0001; HSD OR 3.3, CI 4.5–6.1, *p* < 0.0001), pruritus (itching) (hEDS OR 4.6, CI 7.5–12.4, *p* < 0.0001; HSD OR 6.0, CI 8.3–11.5, *p* < 0001), urticaria (hives) (hEDS OR 6.8, CI 11.5–19.9, *p* < 0.0001; HSD OR 6.4, CI 9.3–13.3, *p* < 0.0001), angioedema (no data), nasal congestion (hEDS OR 29.9, CI 65.2–137.4, *p* < 0.0001; HSD OR 8.6, CI 12.3–17.6, *p* < 0.0001), nasal pruritus (itching) (no data), wheezing (hEDS OR 5.8, CI 17.9–55.3, *p* < 0.0001; HSD OR 4.0, CI 6.3–10.0, *p* < 0.0001), throat swelling (hEDS OR 7.3, CI 13.5–24.3, *p* < 0.0001; HSD OR 3.9, CI 5.7–8.4, *p* < 0.0001), headache (hEDS OR 2.3, CI 3.9–7.1, *p* < 0.0001; HSD OR 3.5, CI 5.0–7.2, *p* < 0.0001), hypotension (hEDS OR 1.8, CI 3.0–4.8, *p* < 0.0001; HSD OR 2.1, CI 3.0–4.3, *p* < 0.0001) and diarrhea (hEDS OR 1.7, CI 2.7–4.1, *p* < 0.0001; HSD OR 2.6, CI 3.5–4.7, *p* < 0.0001) which all had higher ORs in patients with a high MC score compared to low MC score (Fig. 1e).(25) We did not include angioedema or nasal pruritus which were not a part of the questionnaire.

Additionally, we compared total IgE levels in the serum of hEDS/HSD patients with low vs. high MC scores. We found that hEDS/HSD patients with high MC scores had significantly greater serum levels of total IgE than patients with low MC scores (Lo MC *n* = 31, Hi MC *n* = 47, *p* = 0.029) (Fig. 1f), indicating that greater MC activity is related to a higher MC score.

One hypothesis put forward is that MC activation contributes to joint remodeling, leading to weakness and increased hypermobility, resulting in more subluxations and dislocations in hEDS and HSD patients.(26) To investigate this possibility, we examined whether sex differences existed in the level of collagen and other extracellular matrix (ECM) proteins in the serum by ELISA. We found that females with hEDS/HSD had significantly higher serum levels of collagen-4α1 (*n* = 39/sex, *p* < 0.0001), laminin (*n* = 39/sex, *p* = 0.0146), and CCN6 (cellular communication network factor 6) (*n* = 25/sex, *p* = 0.0067) than males (Fig. 1g-i). These findings suggest that females exhibit more MC activation and greater remodeling, which contributes to elevated ECM proteins in the serum, compared to males. Thus, overall, females with hEDS and HSD have indicators of higher MC activity than males.

Out of 21 allergy symptoms and comorbidities examined from the Intake Questionnaire, 5/21 (23.8%) symptoms/comorbidities differed significantly by sex in patients with hEDS, while 10/21 (47.6%) differed by sex in HSD patients (Table 5). As another way to view the data, for hEDS patients 16/21 (76.2%) did not differ by sex, while for HSD 11/21 (52.4%) did not differ by sex (Table 5). Overall, HSD females had more MC-related symptoms/comorbidities than males while most MC symptoms were similar by sex in hEDS patients.

All allergy symptoms/comorbidities with a significant sex difference occurred more often in females than males for both diagnoses. However, the specific MC symptoms that were worse in females with hEDS and HSD differed by diagnosis. Females with hEDS had more drug allergies (Female 61.7%, Male 35.9%, *p* = 0.002), shortness of breath (Female 35.3%, Male 21.7%, *p* = 0.034), urticaria (hives) (Female 21.9%, Male 10.0%, *p* = 0.030), and venom allergies (Female 22.5%, Male 0.0%, *p* < 0.001) (Table 5). In contrast, females with HSD had more environmental allergies (Female 68.0%, Male 56.3%, *p* = 0.023), palpitations (Female 48.8%, Male 28.4%, *p* < 0.001), multiple sensitivities (Female 48.7%, Male 28.4%, *p* < 0.001), rhinitis (Female 48.7%, Male 28.4%, *p* = 0.004), sun sensitivity (Female 32.5%, Male 17.0%, *p* = 0.002), urticaria (hives) (Female 22.5%, Male 6.8%, *p* < 0.001), rash (Female 22.3%, Male 6.8%, *p* < 0.001), and asthma as an adult (Female 22.4%, Male 12.5%, *p* = 0.029) than HSD males (Table 5). Remarkably, none of the issues in hEDS and HSD females were the same, except for hives, which occurred in both. Thus, overall, more females with HSD self-reported allergy symptoms/comorbidities than hEDS females compared to males.

### Neurological symptoms/comorbidities

**Figure 2:**
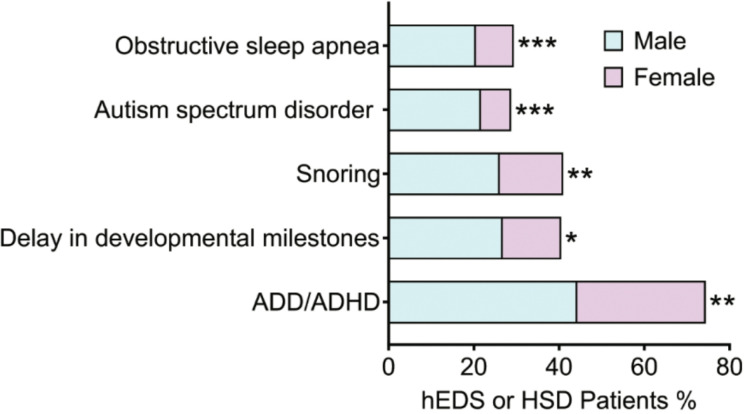
Symptoms and comorbidities increased in males with hEDS or HSD. Only 5 out of 122 (4.4%) symptoms/comorbidities were significantly increased in males compared to females with hEDS or HSD. P-values were obtained using two-tailed Fisher’s exact tests comparing females to males by diagnosis: *, *p* < 0.05; **, *p* < 0.01; ***, *p* < 0.001.

We examined 34 neurological conditions that are listed in Table 6 from greatest to lowest percentage based on female patients with HSD. This is one of only two categories where we found conditions that were self-reported more often in males than females (Fig. 2). Males with hEDS self-reported a delay in developmental milestones (hEDS Female 13.7% hEDS Male 26.8%; *p* = 0.030) and autism spectrum disorder (ASD) (hEDS Female 7.1%, hEDS Male 21.7%; *p* < 0.001) more often than females (Table 6, Fig. 2). In contrast, males with HSD reported attention deficit disorder (ADD)/attention deficit hyperactivity disorder (ADHD) more often than HSD females (HSD Female 30.1%, HSD Male 44.3%; *p* = 0.005) (Table 6, Fig. 2). All other significant sex differences occurred more often in females than males in this category. Notably, except for headache/migraine-related symptoms, females and males with hEDS reported similar levels of most neurological symptoms/comorbidities. In contrast, almost all the most common neurological issues in patients with HSD occurred more often in females (Table 6).

We found that hEDS females self-reported 7/34 (20.6%) neurological conditions more often than males, while HSD females reported 19/34 (55.9%) of these conditions more often than males. Multiple conditions occurred more often in hEDS and HSD females including brain fog (hEDS Female 77.7%, Male 58.3%, *p* < 0.001; HSD Female 79.5%, Male 62.5%, *p* < 0.001), headache (hEDS Female 75.7%, Male 63.3%, *p* = 0.037, HSD Female 73.8%, Male 60.2%, *p* = 0.005), migraine (hEDS Female 58.4%, Male 45.0%, *p* = 0.045, HSD Female 56.5%, Male 36.4%, *p* < 0.001), sense of imbalance (hEDS Female 45.7%, Male 28.3%, *p* = 0.010, HSD Female 54.6%, Male 38.6%, *p* = 0.003), cold intolerance (hEDS Female 49.0%, Male 25.0%, *p* < 0.001, HSD Female 49.2%, Male 28.4%, *p* < 0.001), vertigo (hEDS Female 41.0%, Male 18.3%, *p* < 0.001, HSD Female 41.0%, Male 23.9%, *p* = 0.001), chronic migraine (hEDS Female 30.3%, Male 11.7%, *p* = 0.002, HSD Female 28.6%, Male 11.4%, *p* < 0.001), and neuropathy (hEDS Female 27.5%, Male 13.3%, *p* = 0.018, HSD Female 25.9%, Male 13.6%, *p* = 0.010) (Table 6). The only neurological symptom/comorbidity that occurred more often uniquely in hEDS females was numbness/tingling of extremities (Female 52.3%, Male 36.7%, *p* = 0.021). Neurological symptoms/comorbidities that occurred more often uniquely in HSD females included fibromyalgia (Female 68.5%, Male 50.0%, *p* < 0.001), lightheadedness (Female 65.7%, Male 43.2%, *p* < 0.001), heat intolerance (Female 51.1%, Male 28.4%, *p* < 0.001), palpitations (Female 48.8%, Male 28.4%, *p* < 0.001), multiple sensitivities (i.e., lights, smells, foods, medicine) (Female 48.7%, Male 28.4%, *p* < 0.001), dry eyes (Female 41.0%, Male 25.0%, *p* = 0.003), blurred vision (Female 40.0%, Male 22.7%, *p* = 0.001), autonomic dysfunction (Female 32.3%, Male 17.0%, *p* = 0.003), new daily persistent headache (NDPH) (Female 29.6%, Male 18.2%, *p* = 0.021), and abnormal brain MRI (current or past) (Female 10.5%, Male 3.4%, *p* = 0.031) (Table 6).

### Gastrointestinal symptoms/comorbidities

We examined 19 gastrointestinal (GI) symptoms/comorbidities that are listed from greatest to lowest percentage in Table 7 based on self-reported data from female patients with HSD. All significant sex differences occurred more often in females than males in this category. We found that hEDS females self-reported 5/19 (26.3%) GI conditions more often than males, while HSD females reported 8/19 (42.1%) of these conditions more often than males. Several GI symptoms occurred more often in hEDS and HSD females including nausea (hEDS Female 62.6%, Male 46.7%, *p* = 0.016, HSD Female 64.1%, Male 38.6%, *p* < 0.001), constipation (hEDS Female 61.7%, Male 40.0%, *p* = 0.001, HSD Female 62.2%, Male 31.8%, *p* < 0.001), and vomiting (hEDS Female 34.3%, Male 15.0%, *p* = 0.002, HSD Female 33.9%, Male 18.2%, *p* = 0.002) (Table 7). Dyspepsia (heartburn) (Female 31.5%, Male 16.7%, *p* = 0.017) and hemorrhoids (Female 36.5%, Male 20.0%, *p* = 0.011) occurred uniquely more often in females with hEDS than males. Unique GI symptoms/comorbidities in females with HSD included pain/cramps in lower abdomen (Female 57.3%, Male 35.2%, *p* < 0.001), diarrhea (Female 55.8%, Male 36.4%, *p* < 0.001), bowel cramps (Female 50.9%, Male 39.8%, *p* = 0.041), and irritable bowel syndrome (Female 37.5%, Male 21.6%, *p* = 0.003). None of the GI issues occurred more often in males with hEDS or HSD. Thus, overall, the symptoms/comorbidities that affected most hEDS and HSD patients (> 60%) (i.e., nausea and constipation) occurred more often in females than males.

### Sleep symptoms/comorbidities

We examined 9 sleep symptoms/comorbidities that are listed from greatest to lowest percentage in Table 8 based on female patients with HSD. This is one of only two categories where we found conditions that were self-reported more often in males than females. Males with HSD self-reported more snoring (Female 14.9%, Male 26.1%; *p* = 0.005) and obstructive sleep apnea (Female 8.9%, Male 20.5%; *p* < 0.001) than females with HSD (Table 8, Fig. 2). All other significant sex differences occurred more often in females than males in this category. Sleep issues reported more often in females than males with HSD included insomnia (Female 45.9%, Male 33.0%; *p* = 0.017) and sleep disturbances (Female 42.2%, Male 28.4%; *p* = 0.010) (Table 8). There were no sex differences in any other symptom/comorbidity in this category.

### Psychological conditions and abuse

We did not observe sex differences in most psychological conditions in patients diagnosed with hEDS or HSD (Table 9). The most frequent psychological symptom was anxiety which was self-reported more often in females with HSD than males (Female 73.9%, Male 61.4%; *p* = 0.010) but had no sex difference in patients with hEDS (Female 68.0%, Male 66.7%; *p* = 0.83). The only psychological condition that occurred more often in females for both diagnoses was abuse (hEDS: Female 41.8%, Male 25.0%; *p* < 0.039; HSD: Female 40.0%, Male 24.1%; *p* < 0.001) and sexual abuse (hEDS: Female 23.8%, Male 10.0%; *p* = 0.015; HSD: Female 22.7%, Male 5.7%; *p* < 0.001) (Table 9). Additionally, females with HSD reported more post-traumatic stress disorder (PTSD) (Female 30.3%, Male 11.4%; *p* < 0.001) than males (Table 9).

### Genitourinary symptoms

All genitourinary symptoms examined in this study that were statistically significant were self-reported more often in females with hEDS or HSD compared to males (Table 10). Symptoms/ comorbidities that were self-reported more often in females in both hEDS and HSD included dyspareunia (pain during sexual intercourse) (hEDS Female 31.1%, Male 3.3%; *p* < 0.001; HSD Female 28.1%, Male 4.5%; *p* < 0.001), recurrent urinary tract infections (hEDS Female 30.4%, Male 5.0%; *p* < 0.001; HSD Female 27.4%, Male 3.4%; *p* < 0.001), incontinence (hEDS Female 25.9%, Male 5.0%; *p* < 0.001; HSD Female 23.7%, Male 6.8%; *p* < 0.001), pelvic floor dysfunction (hEDS Female 22.4%, Male 10.0%; *p* = 0.025; HSD Female 18.6%, Male 4.5%; *p* < 0.001), recurrent yeast infections (hEDS Female 19.0%, Male 3.3%; *p* < 0.001; HSD Female 18.0%, Male 0.0%; *p* < 0.001), and bladder prolapse (hEDS Female 8.3%, Male 0.0%; *p* = 0.020; Female 4.9%, Male 0.0%; *p* = 0.033). hEDS females uniquely reported more frequent urination than males (Females 34.4%, Males 20.0%; *p* = 0.024) (Table 10).

## Discussion

This is the first study to analyze sex differences in a large number of symptoms and comorbidities in hEDS and HSD patients. In this study we found that females and males that attended the EDS Clinic shared the same wide range of 122 symptoms/comorbidities. However, females with hEDS and HSD reported more symptoms/comorbidities than males. Interestingly, there were only 5 comorbidities that males reported more often than females including attention deficit disorder (ADD)/ attention deficit hyperactivity disorder (ADHD), delay in developmental milestones, snoring, autism spectrum disorder (ASD), and obstructive sleep apnea (Fig. 2)- conditions with a known male dominance.(27–32) Does that suggest that the symptoms/comorbidities that occur more often in females, such as allergies and migraine, are simply female dominant conditions?(33–35) There are several lines of reasoning to suggest that is not the case. 1) Many of the 122 symptoms/comorbidities in this study do not have a clear reported female dominance, 2) many of the symptoms/comorbidities displayed no sex differences in this study, and 3) females with HSD reported around 2x more symptoms/comorbidities than hEDS females. If the female dominance were due to sex differences in the general population for these symptoms/comorbidities, we would expect hEDS and HSD females to have a similar number of symptoms/comorbidities. Instead, we found that the sex ratio for hEDS was around 10:1 female-to-male while the sex ratio for HSD was around 20:1- a two-fold difference. Additionally, hEDS females reported 31/122 (25.4%) symptoms/comorbidities more often than males while HSD females reported 59/122 (48.4%) more than males- around a two-fold difference. Thus, based on the number of conditions, HSD females had more sex differences in symptoms/comorbidities than hEDS females. From another perspective, hEDS females and males had fewer sex differences, which may suggest greater influence from a genetic variant/s as a driver of disease in hEDS rather than other promoters of disease such as sex hormones.(36)

Another interesting distinction in this study is that for many symptoms/comorbidities the specific issues that occurred more in females than males differed between hEDS and HSD females. For example, although females with hEDS and HSD both self-reported many allergy/mast cell-related symptoms, the specific symptoms that occurred more often than males differed for hEDS (drug allergies, shortness of breath) vs. HSD (environmental allergies, palpitations, multiple sensitivities, rhinitis, etc.) (Table 5), which is consistent with our previous analyses comparing symptoms/comorbidities between patients with hEDS and HSD.(12, 23) Overall, these findings suggest differences in disease burden (i.e., greater number of symptoms/comorbidities) by both sex and diagnosis. Thus, even symptomatic males who attended the Mayo EDS Clinic appear to have a lower disease burden than females. This could, at least in part, explain why fewer hypermobile males seek clinical care for hEDS/HSD in general based on demographic data.(22) Our findings in this study comparing hEDS to HSD females vs. our previous study where males were included gave similar results, except that when males were removed from the analysis in this study, autonomic dysfunction was found more often in HSD rather than hEDS.(12) Otherwise the findings were essentially the same indicating that the small percentage of males in the sample in previous studies did not have a big effect on the data. It is difficult to compare our findings in hEDS patients to the study by Petrucci because they did not analyze data by sex.(22)

An intriguing idea to explain the sex differences in hEDS and HSD is that men have stronger muscle mass from testosterone and so are less likely to be hypermobile; consequently, they are less likely to sublux or dislocate, leading to fewer symptoms. Although this hypothesis makes sense for symptoms such as joint and muscle pain, it is much less clear how muscle strength relates to non-joint-related symptoms like migraine, GI issues, and neurological symptoms. Although this sex difference may partially protect males from hEDS and HSD, another likely cause of sex differences is mast cell activation. MC progenitors originate in the yolk sac and bone marrow and migrate to vascularized tissues/organs in the body where they become long-lived resident MCs and take on characteristics specific to that local environment.(37) MCs are unique among immune cells in that they not only communicate directly with the immune system and local tissue environment but also with the hormone (sex hormones, stress hormones, etc.) and neurological systems.(26, 38, 39) MCs produce neurotransmitters and express neurotransmitter receptors on their cell surface, and many cytokines that they release, such as interleukin (IL)-1β and tumor necrosis factor (TNF), also act as neurotransmitters.(39–43) MCs are located everywhere in the body adjacent to vessels and nerve endings so that they can rapidly transmit signals locally, systemically and via the peripheral and central nervous system including the vagus nerve.(37, 44) MCs can directly activate pain stimuli through the release of TNF, IL-1β and other mediators which all send pain signals to the brain via the nervous system including the vagus nerve.(37, 42–44) MCs also release many mediators (i.e., enzymes, etc.) that are involved in the cycle of ECM remodeling- both breaking down ECM proteins and rebuilding them to produce scar (i.e., fibrosis).(43, 45) Thus, MCs are multi-functional cells that are not limited to IgE-mediated or anaphylactic responses.

Importantly, all these MC functions differ significantly by sex depending on the organ or tissue location.(34, 46) In the heart, cardiac MC degranulation in males drives ECM remodeling that promotes fibrosis and progression to chronic heart failure.(41, 46, 47) Heightened MC activation near sensory neurons has been linked with symptom severity in chronic pain disorders such as irritable bowel syndrome, migraine and fibromyalgia, which all occur more often in females,(34) and are conditions that are prevalent in patients with hEDS and HSD.(12) Higher MC activity in female joints combined with lower muscle strength may exacerbate hypermobility symptoms resulting in higher subluxations and dislocations than the general population in females. This may explain why we observe an increase in many joint and muscle symptoms in females with hEDS or HSD compared to males in this study (Table 3). Another possibility is that HSD females have more activated MCs which could provide an explanation for the increased symptoms/comorbidities observed in HSD females compared to hEDS females.(34)

That MC activation can lead to widespread systemic issues in the skin, GI tract, cardiovascular, respiratory and naso-ocular systems was recognized in 2010 in a manuscript by Akin et al. who proposed a clinical definition of MCAS that was later affirmed by an international panel of experts.(25, 48) For a definition of MCAS the following three criteria must be met: 1. episodic symptoms consistent with MC degranulation involving at least 2 organ systems (i.e., cutaneous, GI, cardiovascular, or respiratory), 2. event-related increase in serum tryptase above the individual’s baseline tryptase to 20% + 2ng/mL, and 3. response of symptoms to therapy with MC-stabilizing agents, drugs directed against MC mediator production, or drugs blocking mediator release or effects of MC-derived mediators.(25, 48) Thus, to receive a diagnosis of MCAS the patient needs to be clinically documented to undergo an anaphylactic type event with the assumption that the patient typically has a low/lower baseline tryptase. However, the presumed mechanism behind MC activation in patients with hEDS and HSD suggests that patients have daily, chronic MC activation which would create high baselines that likely limit the ability to mount an acute event. Furthermore, Valent et al. stated that the chronic systemic symptoms must be associated only with MCAS and not attributed to any other medical condition.(25) This statement essentially eliminates hEDS and HSD patients from being candidates for this diagnosis and may inhibit clinical immunologists from pursuing testing.

Because of this clinical gap, we (Drs. Fairweather and Bruno) developed a self-reported MC score with the goal of capturing widespread MC activation symptoms that include, but are not limited to, MCAS, tryptase mutations and mastocytosis.(25, 49) The symptoms included in the MC score encompass categories of MC issues such as asthma/history of asthma, environmental, venom, food and drug allergies, atopy, eczema, rhinitis, MCAS and tryptase mutations. These are self-reported symptoms and so may not be accurately diagnosed; however, answers to these self-reported questions should enable identification of widespread issues if they exist. Using this novel MC score, we are the first to examine whether widespread MC activation in patients with hEDS and HSD differs by sex. We provide internal validation of the MC score in several ways: 1) by showing significantly increased odds ratios for high vs. low MC scores for MC score-associated symptoms, 2) by showing significantly increased odds ratios for high vs. low MC scores for MC activation symptoms that comprise part of the MCAS diagnostic criteria (Fig. 1e),(25, 48) and 3) by showing that patients with a high MC score have significantly higher levels of total IgE in their serum than patients with a low MC score (Fig. 1f). We plan to further validate the MC score in hEDS and HSD patients from other sites in the future.

In this study we found that MCAS was self-reported in around 5–17% of hEDS/HSD patients, but these percentages may not include verified cases of MCAS but possibly a patient’s perception of having this condition. A recent study of 703 patients referred for MC activation symptoms found that only 4.4% had MCAS according to diagnostic criteria.(50) Only ≤ 1% of patients in our study self-reported that they had a tryptase mutation, which is part of the diagnostic criteria for hereditary alpha tryptasemia (HAT).(51) The diagnosis of HAT includes demonstrating an increased gene copy number of *TPSAB1* encoding for alpha tryptase and elevated serum tryptase levels.(51) MCAS and HAT are considered MC activation disorders (MCAD) along with systemic mastocytosis.(25, 49) Brock et al. describes that MC activation disease (MCAD) should include widespread MC-related symptoms in many different organ systems such as GI, cardiovascular, respiratory, skin, neurological, musculoskeletal, etc. and verified MCAS,(9) but did not create a measurable MC activation score. Note that MC activation disorders vs. MC activation disease have the same abbreviation (i.e., MCAD) but different definitions depending on the author.(9, 49) Most of the widespread MC-related symptoms listed by Brock et al.(9) and Valent et al.(25) are included in our Intake Questionnaire and were reported more often in females with hEDS and HSD compared to males. Additionally, our MC score indicates widespread MC activation issues, which we found were reported more often in HSD females but similarly between hEDS females and males. Thus, the MC score questionnaire we developed is a fast and simple method to assess widespread MC activation issues in large numbers of patients without lengthy and expensive clinical testing.

This study revealed several other interesting findings. A high percentage of hEDS and HSD patients reported anxiety and depression (around 60% in this study) similar to previous reports,(12, 23) yet except for abuse there were few sex differences in psychological symptoms/comorbidities in this study. Males who attended the EDS Clinic were slightly younger than females. Surprisingly, almost all teeth and jaw symptoms were worse in females with HSD; however, only a high or narrow palate occurred more often in females with hEDS, which is part of the diagnostic criteria for hEDS.(4) It was also remarkable that none of the MC-related symptoms in hEDS and HSD females were the same, except for hives, which occurred in both. Also interesting related to neurological symptoms/comorbidities was that females of both diagnoses had greater brain fog, headache, migraine, sense of imbalance, vertigo and chronic migraine- which we reported previously as similar between diagnoses.(12) In contrast, unique to HSD females was greater fibromyalgia, light-headedness, heat intolerance, palpitations, multiple sensitivities, dry eyes, and blurred vision- all symptoms of autonomic dysfunction,(52) and which comprise part of the diagnostic criteria for fibromyalgia.(53) Thus, our findings suggest that females with HSD have more autonomic dysfunction. However, these findings require confirmation using medical record extraction and/or physician validation in future studies.

There are several limitations to this study. The retrospective design of the study precludes establishing causal relationships. The site of the study is a tertiary care center and findings from patients in this study may not be generalizable to other regions of the US or world. Symptoms/comorbidities were self-reported and not validated through another method. Future studies are needed to examine whether the key findings of this study can be verified when medical records are examined. A strength of the study is that hEDS and HSD patients were diagnosed using the most recent 2017 criteria by physician experts. An additional strength of the study is the large study population which provided the ability to study a small cohort of males. To our knowledge, this is the first study to analyze sex differences in a large number of symptoms/comorbidities in males and females with hEDS and HSD.

### Perspectives and Significance

The ability to study sex differences was made possible by large numbers of well-characterized patients. Overall, we found large sex differences in the number of patients attending the EDS Clinic that were diagnosed with hEDS and HSD and the self-reported burden of symptoms/comorbidities, with females more greatly affected. Like our previous studies,(12, 23) females with HSD reported more symptoms/comorbidities than females with hEDS. In contrast, only 5/122 (4.1%) of symptoms/comorbidities occurred more often in males than females, which may be due to an increased prevalence of these issues (i.e., ASD) in males in the general population rather than an EDS-driven pathology. Our findings suggest a difference in disease burden (i.e., number of symptoms/comorbidities) by sex and diagnosis. Elevated MC symptoms in females may drive symptomology indicated by elevated serum extracellular matrix proteins in females.

## Conclusions

In conclusion, we found that females with hEDS and HSD self-reported more symptoms/ comorbidities than males with a sex ratio of around 10:1 and 20:1 female-to-male, respectively. Gaining a better understanding of sex differences in patients with hEDS and HSD may improve patient care, optimize a personalized approach to therapy, and provide mechanistic insights into the pathogenesis of disease.

## Supplementary Material

None.

## Figures and Tables

**Table 1:** Demographics of female vs. male patients diagnosed at the EDS Clinic (*n* = 2,451)

	hEDS^a^ Female (*n* = 575)	hEDS Male (*n* = 60)	*P* value^b^	HSD Female (*n* = 1,728)	HSD Male (*n* = 88)	*P* value^b^
*Median age (SD)*	35.3 (11.8)	28.3 (9.4)	**< 0.001** ^c^	34.4 (12.1)	30.6 (13.3)	**0.004**
*Race*						
American Indian/ Alaska native	9 (1.6)	1 (1.7)	0.95	26 (1.5)	2 (2.3)	0.57
Asian	9 (1.6)	0 (0.0)	0.33	34 (2.0)	0 (0.0)	0.18
Black or African American	19 (3.3)	2 (3.3)	0.99	42 (2.4)	2 (2.3)	0.92
Native Hawaii/ Pacific Islander	1 (0.2)	0 (0.0)	0.75	4 (0.2)	0 (0.0)	0.65
White	514 (89.4)	54 (90.0)	0.88	1619 (93.7)	85 (96.6)	0.27
Other	17 (3.0)	1 (1.7)	0.57	46 (2.7)	2 (2.3)	0.82
Unknown/ not disclosed	7 (1.2)	2 (3.3)	0.19	16 (0.9)	0 (0.0)	0.36
*Ethnicity*			0.80			0.45
Hispanic/ Latino	39 (7.1)	3 (5.3)		138 (8.1)	4 (4.6)	
Not Hispanic/ Latino	493 (90.3)	53 (93.0)		1531 (89.4)	80 (92.0)	
Choose not to disclose/unknown	14 (2.6)	1 (1.8)		43 (2.5)	3 (3.4)	
*Highest level of education*			0.07			**0.001**
Some high school	10 (1.9)	5 (9.1)		68 (4.0)	12 (13.8)	
High school/GED	42 (8.0)	7 (12.7)		145 (8.5)	9 (10.3)	
Some college	118 (22.5)	13 (23.6)		358 (21.0)	21 (24.1)	
Trade school	21 (4.0)	2 (3.6)		77 (4.5)	4 (4.6)	
Associates	66 (12.6)	6 (10.9)		194 (11.4)	6 (6.9)	
Bachelors	156 (29.7)	14 (25.5)		515 (30.2)	19 (21.8)	
Masters	79 (15.0)	4 (7.3)		249 (14.6)	8 (9.2)	
Professional/ doctorate	29 (5.5)	4 (7.3)		91 (5.3)	7 (8.0)	
Not disclosed	4 (0.8)	0 (0.0)		9 (0.5)	1 (1.1)	

a (*n*, %),

b Fisher’s exact test or Kruskal-Wallis rank sum compares differences in categorical variables between groups. Student’s *t* test or Mann-Whitney rank test compares the differences between continuous variables between groups.

c Bold indicates significant value.

**Table 2:** Self-reported environmental exposures (*n* = 2,451)

	hEDS^a^ Female (*n* = 575)	hEDS Male (*n* = 60)	*P* value^b^	HSD Female (*n* = 1,728)	HSD Male (*n* = 88)	*P* value^b^
*BMI*			0.80			0.14
Mean (SD)	24.9 (5.4)	25.1 (4.9)		26.3 (5.6)	25.3 (5.4)	
Range	23.5 (16.6, 39.7)	24.5 (16.6, 36.6)		25.2 (16.6, 40.0)	24.2 (17.5, 39.9)	
*Smoking history*			**0.005** ^c^			0.16
Yes-Currently	55 (10.1)	10 (17.5)		146 (8.5)	12 (13.8)	
Yes-Past	108 (19.9)	6 (10.5)		282 (16.5)	9 (10.3)	
No	379 (69.7)	39 (68.4)		1271 (74.2)	66 (75.9)	
Unknown	2 (0.4)	2 (3.5)		13 (0.8)	0 (0.0)	
*Number of cigarettes smoked/day*			0.18			0.61
1–5	28 (17.4)	1 (6.2)		83 (19.4)	1 (4.8)	
6–10	19 (11.8)	2 (12.5)		49 (11.4)	2 (9.5)	
10–20	18 (11.2)	0 (0.0)		46 (10.7)	3 (14.3)	
> 20	11 (6.8)	0 (0.0)		16 (3.7)	1 (4.8)	
Unknown	25 (15.5)	2 (12.5)		68 (15.9)	3 (14.3)	
Vaping	60 (37.3%)	11 (68.8%)		166 (38.8%)	11 (52.4%)	
*Alcohol consumption*			0.09			**0.007**
Yes-Currently	292 (53.7)	24 (42.1)		881 (51.5)	39 (44.8)	
Yes-Past	153 (28.1)	16 (28.1)		444 (25.9)	15 (17.2)	
No	94 (17.3)	15 (26.3)		382 (22.3)	33 (37.9)	
Unknown	5 (0.9)	2 (3.5)		5 (0.3)	0 (0.0)	
*Number of alcoholic drinks consumed on average/ week*			0.61			0.60
0–1	168 (37.9)	10 (25.0)		536 (40.5)	25 (46.3)	
2–3	86 (19.4)	10 (25.0)		216 (16.3)	6 (11.1)	
4–7	34 (7.7)	4 (10.0)		111 (8.4)	5 (9.3)	
7+	15 (3.4)	2 (5.0)		37 (2.8)	3 (5.6)	
1–2 drinks/ month	121 (27.3)	11 (27.5)		394 (29.7)	13 (24.1)	
Unknown	19 (4.3)	3 (7.5)		31 (2.3)	2 (3.7)	
*Alcohol exposure before birth*			0.44			0.36
Yes	15 (2.9)	1 (1.9)		34 (2.0)	0 (0.0)	
No	461 (87.6)	45 (83.3)		1492 (87.4)	76 (87.4)	
Unknown	50 (9.5)	8 (14.8)		181 (10.6)	11 (12.6)	
*Illicit drug use*			**0.036**			0.08
Yes-Currently	14 (2.6)	5 (8.8)		59 (3.4)	6 (6.9)	
Yes-Past	42 (7.7)	7 (12.3)		104 (6.1)	8 (9.2)	
No	458 (84.0)	43 (75.4)		1459 (85.2)	72 (82.8)	
Unknown/ choose not to disclose	31 (5.7)	2 (3.5)		90 (5.3)	1 (1.1)	
*Exposure to drugs as a baby*			0.43			0.76
Yes	14 (2.6)	2 (3.6)		45 (2.6)	2 (2.3)	
No	460 (85.2)	44 (78.6)		1475 (86.1)	73 (83.9)	
Unknown	66 (12.2)	10 (17.9)		193 (11.3)	12 (13.8)	

a (*n*, %),

b Fisher’s exact test compares differences in categorical variables between groups. Student’s *t* test or Mann-Whitney rank test compares the differences between continuous variables between groups.

c Bold indicates significant value.

**Table 3:** Joint symptoms/comorbidities in patients with hEDS or HSD (*n* = 2,451)

	hEDS^a^ Female (n = 575)	hEDS Male (n = 60)	P value^b^	HSD Female (n = 1,728)	HSD Male (n = 88)	P value^b^
Joint pain	519 (90.3)	46 (76.7)	**0.001** ^c^	1635 (94.6)	77 (87.5)	**0.005**
Subluxations	448 (77.9)	43 (71.7)	0.27	1320 (76.4)	57 (64.8)	**0.013**
Sprains	432 (75.1)	30 (50.0)	**< 0.001**	1198 (69.3)	44 (50.0)	**< 0.001**
Easy bruising	326 (56.7)	14 (23.3)	**< 0.001**	1117 (64.6)	25 (28.4)	**< 0.001**
Muscle weakness	278 (48.3)	24 (40.0)	0.22	975 (56.4)	45 (51.1)	0.33
TMJ symptoms^d^	244 (42.4)	22 (36.7)	0.39	847 (49.0)	24 (27.3)	**< 0.001**
Dislocations	231 (40.2)	25 (41.7)	0.82	525 (30.4)	27 (30.7)	0.95
No joint issues	6 (1.0)	4 (6.7)	**< 0.001**	28 (1.6)	3 (3.4)	0.21

a (*n*, %),

b Fisher’s exact test compares differences in categorical variables between groups.

c Bold indicates significant value.

d Abbreviations: TMJ, temporomandibular joint symptoms.

**Table 4:** Teeth and jaw symptoms/comorbidities self-reported in patients with hEDS or HSD (*n* = 2,451)

	hEDS^a^ Female (*n* = 575)	hEDS Male (*n* = 60)	*P* value^b^	HSD Female (*n* = 1,728)	HSD Male (*n* = 88)	*P* value^b^
Braces needed as a child or adult	393 (68.3)	39 (65.0)	0.60	1135 (65.7)	46 (52.3)	**0.010** ^c^
Cavities	326 (56.7)	29 (48.3)	0.21	934 (54.1)	36 (40.9)	**0.016**
Bleeding gums	284 (49.4)	33 (55.0)	0.41	861 (49.8)	24 (27.3)	**< 0.001**
TMJ symptoms^d^	244 (42.4)	22 (36.7)	0.39	847 (49.0)	24 (27.3)	**< 0.001**
Teeth being pulled out/ overcrowding	279 (48.5)	22 (36.7)	0.08	703 (40.7)	26 (29.5)	**0.038**
High or narrow palate	180 (31.3)	11 (18.3)	**0.037**	444 (25.7)	11 (12.5)	**0.005**
Brittle teeth	104 (18.1)	11 (18.3)	0.96	234 (13.5)	6 (6.8)	0.07
None	32 (5.6)	3 (5.0)	0.86	142 (8.2)	17 (19.3)	**< 0.001**

a (*n*, %),

b Fisher’s exact test compares differences in categorical variables between groups.

c Bold indicates significant value.

d Abbreviations: TMJ, temporomandibular joint symptoms.

**Table 5:** Allergy-related symptoms/comorbidities in patients with hEDS or HSD (*n* = 2,451)

	hEDS^a^ Female (*n* = 575)	hEDS Male (*n* = 60)	*P* value^b^	HSD Female (*n* = 1,728)	HSD Male (*n* = 88)	*P* value^b^
MC score, mean (SD)	3.3 (2.1)	2.7 (1.8)	0.11	3.1 (1.9)	2.4 (1.9)	**0.0003** ^c^
Allergy/atopy^d^	164 (79.6)	15 (60.0)	0.09	403 (77.5)	15 (75.0)	0.96
Environmental allergies	368 (68.5)	38 (67.9)	0.92	1165 (68.0)	49 (56.3)	**0.023**
Drug allergies	230 (61.7)	14 (35.9)	**0.002**	798 (55.8)	38 (50.0)	0.32
Palpitations	261 (45.4)	20 (33.3)	0.07	844 (48.8)	25 (28.4)	**< 0.001**
Multiple sensitivities	243 (42.3)	25 (41.7)	0.93	842 (48.7)	25 (28.4)	**< 0.001**
Rhinitis	226 (42.2)	19 (33.9)	0.23	738 (43.1)	24 (27.6)	**0.004**
Chest discomfort	210 (36.5)	24 (40.0)	0.59	727 (42.1)	38 (43.2)	0.84
Shortness of breath	203 (35.3)	13 (21.7)	**0.034**	678 (39.2)	26 (29.5)	0.07
Food allergies	128 (34.3)	10 (25.6)	0.28	502 (35.1)	19 (25.0)	0.07
Sun sensitivity	147 (25.6)	15 (25.0)	0.92	561 (32.5)	15 (17.0)	**0.002**
Eczema	98 (26.3)	15 (38.5)	0.10	393 (27.5)	22 (28.9)	0.78
Hives	126 (21.9)	6 (10.0)	**0.030**	388 (22.5)	6 (6.8)	**< 0.001**
Rash	121 (21.0)	7 (11.7)	0.09	385 (22.3)	6 (6.8)	**< 0.001**
Oral ulcers	88 (15.3)	8 (13.3)	0.68	342 (19.8)	14 (15.9)	0.37
Venom allergies	84 (22.5)	0 (0.0)	**< 0.001**	233 (16.3)	6 (7.9)	0.05
MCAS	92 (17.1)	6 (10.7)	0.22	190 (11.1)	5 (5.7)	0.12
Wheezing	56 (9.7)	6 (10.0)	0.95	184 (10.6)	8 (9.1)	0.64
Tryptase mutation	5 (0.9)	0 (0.0)	0.47	4 (0.2)	1 (1.1)	0.11
Asthma history						
Asthma as a child	175 (30.4)	16 (26.7)	0.54	518 (30.0)	28 (31.8)	0.71
Asthma as an adult	133 (23.1)	8 (13.3)	0.08	387 (22.4)	11 (12.5)	**0.029**
No asthma	263 (45.7)	30 (50.0)	0.53	900 (52.1)	53 (60.2)	0.14
Asthma unknown	40 (7.0)	7 (11.7)	0.18	141 (8.2)	6 (6.8)	0.65

a (*n*, %),

b Fisher’s exact test compares differences in categorical variables between groups.

c Bold indicates significant value.

d Except for MC score and asthma history, order based on highest percentage in HSD females.

**Table 6:** Neurologic symptoms/comorbidities in patients with hEDS or HSD (*n* = 2,451)

	hEDS^a^ Female (*n* = 575)	hEDS Male (*n* = 60)	*P* value^b^	HSD Female (*n* = 1,728)	HSD Male (*n* = 88)	*P* value^b^
Brain fog^c^	447 (77.7)	35 (58.3)	**< 0.001** ^d^	1374 (79.5)	55 (62.5)	**< 0.001**
Headache	435 (75.7)	38 (63.3)	**0.037**	1276 (73.8)	53 (60.2)	**0.005**
Fibromyalgia	341 (59.3)	30 (50.0)	0.16	1184 (68.5)	44 (50.0)	**< 0.001**
Lightheadedness	328 (57.0)	33 (55.0)	0.76	1135 (65.7)	38 (43.2)	**< 0.001**
Numbness/tingling of extremities	301 (52.3)	22 (36.7)	**0.021**	1086 (62.8)	48 (54.5)	0.12
Migraine	336 (58.4)	27 (45.0)	**0.045**	976 (56.5)	32 (36.4)	**< 0.001**
Sense of imbalance	263 (45.7)	17 (28.3)	**0.010**	944 (54.6)	34 (38.6)	**0.003**
Heat intolerance	248 (43.1)	24 (40.0)	0.64	883 (51.1)	25 (28.4)	**< 0.001**
Tinnitus	297 (51.7)	28 (46.7)	0.46	862 (49.9)	40 (45.5)	0.42
Cold intolerance	282 (49.0)	15 (25.0)	**< 0.001**	850 (49.2)	25 (28.4)	**< 0.001**
Palpitations	261 (45.4)	20 (33.3)	0.07	844 (48.8)	25 (28.4)	**< 0.001**
Multiple sensitivities	243 (42.3)	25 (41.7)	0.93	842 (48.7)	25 (28.4)	**< 0.001**
Ringing in the ears	243 (42.3)	25 (41.7)	0.93	808 (46.8)	36 (40.9)	0.28
Vertigo	236 (41.0)	11 (18.3)	**< 0.001**	709 (41.0)	21 (23.9)	**0.001**
Dry eyes	208 (36.2)	17 (28.3)	0.23	708 (41.0)	22 (25.0)	**0.003**
Blurred vision	217 (37.7)	18 (30.0)	0.24	691 (40.0)	20 (22.7)	**0.001**
Increased sweating	180 (31.3)	20 (33.3)	0.75	661 (38.3)	30 (34.1)	0.43
Dry mouth	175 (30.4)	16 (26.7)	0.54	606 (35.1)	22 (25.0)	0.05
Autonomic dysfunction	214 (37.2)	16 (26.7)	0.11	558 (32.3)	15 (17.0)	**0.003**
ADD/ADHD ^e^	159 (27.7)	22 (36.7)	0.14	520 (30.1)	39 (44.3)	**0.005**
New daily persistent headache	166 (28.9)	11 (18.3)	0.08	512 (29.6)	16 (18.2)	**0.021**
Chronic migraine	174 (30.3)	7 (11.7)	**0.002**	495 (28.6)	10 (11.4)	**< 0.001**
Neuropathy	158 (27.5)	8 (13.3)	**0.018**	448 (25.9)	12 (13.6)	**0.010**
Severe illnesses in childhood	149 (27.6)	18 (32.1)	0.76	435 (25.4)	22 (25.3)	0.42
Hearing difficulties	127 (22.1)	9 (15.0)	0.20	413 (23.9)	21 (23.9)	1.00
Cluster headache	76 (13.2)	7 (11.7)	0.74	273 (15.8)	12 (13.6)	0.59
Delay in developmental milestones	74 (13.7)	15 (26.8)	**0.030**	212 (12.4)	14 (16.1)	0.56
Dyslexia	56 (9.7)	4 (6.7)	0.44	192 (11.1)	10 (11.4)	0.94
Abnormal brain MRI (current or past)	84 (14.6)	4 (6.7)	0.09	182 (10.5)	3 (3.4)	**0.031**
Taste (loss/change)	47 (8.2)	5 (8.3)	0.97	182 (10.5)	7 (8.0)	0.44
Autism spectrum disorder (ASD)	41 (7.1)	13 (21.7)	**< 0.001**	149 (8.6)	7 (8.0)	0.83
Intracranial hypertension	30 (5.2)	2 (3.3)	0.53	67 (3.9)	2 (2.3)	0.44
Chiari malformation	21 (3.7)	2 (3.3)	0.90	59 (3.4)	3 (3.4)	1.00
CSF leak	20 (3.5)	3 (5.0)	0.55	41 (2.4)	3 (3.4)	0.54
Other	99 (17.2)	9 (15.0)	0.66	318 (18.4)	16 (18.2)	0.96
Unknown	3 (0.5)	1 (1.7)	0.29	26 (1.5)	1 (1.1)	0.78
No issues	5 (0.9)	6 (10.0)	**< 0.001**	41 (2.4)	10 (11.4)	**< 0.001**

a (*n*, %),

b Fisher’s exact test compares differences in categorical variables between groups.

c Order based on highest percentage in HSD females.

d Bold indicates significant value.

e Abbreviations: ADD; attention deficit disorder; ADHD attention deficit hyperactivity disorder; ASD, autism spectrum disorder; CSF, cerebral spinal fluid; MRI, magnetic resonance imaging.

**Table 7:** Gastrointestinal symptoms/comorbidities in patients with hEDS or HSD (*n* = 2,451)

	hEDS^a^ Female (*n* = 575)	hEDS Male (*n* = 60)	*P* value^b^	HSD Female (*n* = 1,728)	HSD Male (*n* = 88)	*P* value^b^
Nausea^c^	360 (62.6)	28 (46.7)	**0.016** ^d^	1107 (64.1)	34 (38.6)	**< 0.001**
Constipation	355 (61.7)	24 (40.0)	**0.001**	1074 (62.2)	28 (31.8)	**< 0.001**
Pain/cramps in lower abdomen	281 (48.9)	26 (43.3)	0.41	991 (57.3)	31 (35.2)	**< 0.001**
Diarrhea	311 (54.1)	31 (51.7)	0.72	964 (55.8)	32 (36.4)	**< 0.001**
Bowel cramps	253 (44.0)	24 (40.0)	0.55	880 (50.9)	35 (39.8)	**0.041**
GERD (reflux)^e^	242 (42.1)	20 (33.3)	0.19	762 (44.1)	37 (42.0)	0.70
Loss of appetite	197 (34.3)	19 (31.7)	0.69	669 (38.7)	25 (28.4)	0.05
Irritable bowel syndrome (IBS)	203 (35.3)	16 (26.7)	0.18	648 (37.5)	19 (21.6)	**0.003**
Dyspepsia (heartburn)	181 (31.5)	10 (16.7)	**0.017**	640 (37.0)	26 (29.5)	0.16
Frequent loose stools	191 (33.2)	25 (41.7)	0.19	612 (35.4)	26 (29.5)	0.26
Vomiting	197 (34.3)	9 (15.0)	**0.002**	585 (33.9)	16 (18.2)	**0.002**
Hemorrhoids	210 (36.5)	12 (20.0)	**0.011**	507 (29.3)	20 (22.7)	0.18
Gastroparesis	113 (19.7)	12 (20.0)	0.95	347 (20.1)	12 (13.6)	0.14
Fissure (tear in anus)	83 (14.4)	7 (11.7)	0.56	210 (12.2)	7 (8.0)	0.24
Rectal prolapse	46 (8.0)	5 (8.3)	0.93	84 (4.9)	2 (2.3)	0.26
Fecal incontinence (leakage of feces)	42 (7.3)	2 (3.3)	0.25	85 (4.9)	3 (3.4)	0.52
Barrett’s esophagus	9 (1.6)	1 (1.7)	0.95	37 (2.1)	2 (2.3)	0.93
Ulcerative colitis	13 (2.3)	1 (1.7)	0.76	28 (1.6)	3 (3.4)	0.21
Crohn’s disease	9 (1.6)	0 (0.0)	0.33	18 (1.0)	2 (2.3)	0.28
Other	94 (16.3)	9 (15.0)	0.79	285 (16.5)	9 (10.2)	0.12
Unknown	11 (1.9)	6 (10.0)	**< 0.001**	34 (2.0)	1 (1.1)	0.58
No issues	21 (3.7)	4 (6.7)	0.25	96 (5.6)	18 (20.5)	**< 0.001**

a (*n*, %),

b Fisher’s exact test compares differences in categorical variables between groups.

c Order based on highest percentage in HSD females.

d Bold indicates significant value.

e Abbreviations: GERD, gastroesophageal reflux disease.

**Table 8:** Sleep symptoms/comorbidities in patients with hEDS or HSD (*n* = 2,451)

	hEDS^a^ Female (*n* = 575)	hEDS Male (*n* = 60)	*P* value^b^	HSD Female (*n* = 1,728)	HSD Male (*n* = 88)	*P* value^b^
Difficulty falling asleep and staying asleep	318 (55.3)	30 (50.0)	0.43	1118 (64.7)	49 (55.7)	0.09
Insomnia	254 (44.2)	26 (43.3)	0.90	793 (45.9)	29 (33.0)	**0.017**
Sleep disturbances	240 (41.7)	23 (38.3)	0.61	730 (42.2)	25 (28.4)	**0.010**
Restless leg syndrome	135 (23.5)	17 (28.3)	0.40	447 (25.9)	18 (20.5)	0.26
Idiopathic hypersomnia	97 (16.9)	8 (13.3)	0.48	349 (20.2)	12 (13.6)	0.13
Snoring	85 (14.8)	7 (11.7)	0.51	258 (14.9)	23 (26.1)	**0.005**
Obstructive sleep apnea	49 (8.5)	6 (10.0)	0.70	154 (8.9)	18 (20.5)	**< 0.001**
Narcolepsy	33 (5.7)	2 (3.3)	0.44	75 (4.3)	2 (2.3)	0.35
Parasomnia	22 (3.8)	1 (1.7)	0.39	58 (3.4)	1 (1.1)	0.25
Other	48 (8.3)	5 (8.3)	1.00	167 (9.7)	8 (9.1)	0.86
Unknown	50 (8.7)	4 (6.7)	0.59	145 (8.4)	8 (9.1)	0.82
No issues	56 (9.7)	11 (18.3)	**0.039**	202 (11.7)	18 (20.5)	**0.014**

a (*n*, %),

b Fisher’s exact test compares differences in categorical variables between groups.

c Order based on highest percentage in HSD females.

d Bold indicates significant value.

**Table 9:** Psychological symptoms/comorbidities in patients with hEDS or HSD (*n* = 2,451)

	hEDS^a^ Female (*n* = 575)	hEDS Male (*n* = 60)	*P* value^b^	HSD Female (*n* = 1,728)	HSD Male (*n* = 88)	*P* value^b^
Anxiety^c^	391 (68.0)	40 (66.7)	0.83	1277 (73.9)	54 (61.4)	**0.010** ^d^
Depression	327 (56.9)	35 (58.3)	0.83	1065 (61.6)	49 (55.7)	0.26
Depressed mood	218 (37.9)	30 (50.0)	0.07	848 (49.1)	43 (48.9)	0.97
Nervousness	215 (37.4)	27 (45.0)	0.25	815 (47.2)	38 (43.2)	0.46
Abuse	228 (41.8)	14 (25.0)	**0.039**	685 (40.0)	21 (24.1)	**< 0.001**
Emotional/verbal abuse	180 (31.3)	12 (20.0)	0.07	559 (32.3)	20 (22.7)	0.06
PTSD^e^	164 (28.5)	13 (21.7)	0.26	524 (30.3)	10 (11.4)	**< 0.001**
Sexual abuse	137 (23.8)	6 (10.0)	**0.015**	392 (22.7)	5 (5.7)	**< 0.001**
Physical abuse	121 (21.0)	8 (13.3)	0.16	357 (20.7)	12 (13.6)	0.11
Obsessive-compulsive disorder (OCD)	116 (20.2)	6 (10.0)	0.06	298 (17.2)	16 (18.2)	0.82
Eating disorders	77 (13.4)	4 (6.7)	0.14	249 (14.4)	7 (8.0)	0.09
Body dysmorphic disorder	34 (5.9)	6 (10.0)	0.22	152 (8.8)	8 (9.1)	0.92
Bipolar	31 (5.4)	3 (5.0)	0.90	110 (6.4)	3 (3.4)	0.26
Personality disorder	13 (2.3)	1 (1.7)	0.76	51 (3.0)	4 (4.5)	0.40
Conversion disorder	8 (1.4)	2 (3.3)	0.25	24 (1.4)	2 (2.3)	0.50
Schizophrenia	0 (0.0)	0 (0.0)	-	4 (0.2)	0 (0.0)	0.65
Other	31 (5.4)	3 (5.0)	0.90	93 (5.4)	4 (4.5)	0.73
Unknown	15 (2.6)	5 (8.3)	**0.016**	42 (2.4)	5 (5.7)	0.06
No issues	89 (15.5)	7 (11.7)	0.43	259 (15.0)	23 (26.1)	**0.005**

a (*n*, %),

b Fisher’s exact test compares differences in categorical variables between groups.

c Order based on highest percentage in HSD females.

d Bold indicates significant value.

e Abbreviations: PTSD; post-traumatic stress disorder.

**Table 10:** Genitourinary symptoms/comorbidities in patients with hEDS or HSD (*n* = 2,451)

	hEDS^a^ Female (*n* = 575)	hEDS Male (*n* = 60)	*P* value^b^	HSD Female (*n* = 1,728)	HSD Male (*n* = 88)	*P* value^b^
Frequent urination^c^	198 (34.4)	12 (20.0)	**0.024** ^d^	644 (37.3)	27 (30.7)	0.21
Dyspareunia (pain during sexual intercourse	179 (31.1)	2 (3.3)	**< 0.001**	486 (28.1)	4 (4.5)	**< 0.001**
Recurrent urinary tract infections	175 (30.4)	3 (5.0)	**< 0.001**	473 (27.4)	3 (3.4)	**< 0.001**
Incontinence (urine leakage)	149 (25.9)	3 (5.0)	**< 0.001**	409 (23.7)	6 (6.8)	**< 0.001**
Pelvic floor dysfunction	129 (22.4)	6 (10.0)	**0.025**	322 (18.6)	4 (4.5)	**< 0.001**
Recurrent yeast infections	109 (19.0)	2 (3.3)	**0.002**	311 (18.0)	0 (0.0)	**< 0.001**
Pelvic floor spasms	70 (12.2)	4 (6.7)	0.21	176 (10.2)	2 (2.3)	**0.015**
Interstitial cystitis (inflammation of the bladder)/ painful bladder syndrome	59 (10.3)	2 (3.3)	0.08	150 (8.7)	1 (1.1)	**0.012**
Bladder prolapse	48 (8.3)	0 (0.0)	**0.020**	85 (4.9)	0 (0.0)	**0.033**
Other	75 (13.0)	2 (3.3)	**0.028**	227 (13.1)	9 (10.2)	0.43
Unknown	37 (6.4)	11 (18.3)	**< 0.001**	165 (9.5)	6 (6.8)	0.39
No issues	98 (17.0)	32 (53.3)	**< 0.001**	325 (18.8)	63 (71.6)	**< 0.001**

a (*n*, %),

b Fisher’s exact test compares differences in categorical variables between groups.

c Order based on highest percentage in HSD females.

d Bold indicates significant value.

## Data Availability

The datasets generated and/or analyzed during the current study are available in the [NAME] repository, [PERSISTENT WEB LINK TO DATASETS]. (Note: The link to data will be provided prior to publication.)

## Highlights

1. More females than males attended the EDS Clinic with a sex ratio of around 10:1 in hEDS and 20:1 in HSD.
2. Females with hEDS (25%) and HSD (48%) reported more of 122 symptoms/comorbidities than males (4%).
3. Females with HSD reported around 2x more symptoms/comorbidities than hEDS females.
4. Females reported more mast cell-related symptoms/comorbidities than males.
5. Aside from abuse, few sex differences existed in psychological issues.
